# A Cross‐Sectional Study of Prescribed Sleep Medication Use and Its Clinical and Demographic Factors Among Young People Receiving Mental Health Crisis Interventions in Sydney, Australia

**DOI:** 10.1111/jcap.70071

**Published:** 2026-08-06

**Authors:** Md Nazmul Huda, Hasan Jamali, Mobarrat Monir, Avick Rahman, Anne‐Marie Schlesinger, Ingrid Bartrop, Giles Barton, Alvira Hurst, Craig Andrews, Valsamma Eapen, Rajeev Jairam

**Affiliations:** ^1^ Infant, Child and Adolescent Mental Health Service, Mental Health Department South‐Western Sydney Local Health District Sydney NSW Health Australia; ^2^ Discipline of Psychiatry and Mental Health, School of Clinical Medicine University of New South Wales Sydney Australia; ^3^ Academic Unit of Infant Child and Adolescent Psychiatry Services, Ingham Institute for Applied Medical Research, Liverpool Sydney Australia; ^4^ Department of Psychological Sciences Swinburne University of Technology Melbourne Australia; ^5^ School of Medicine Western Sydney University, Campbelltown Sydney Australia

**Keywords:** children and adolescents, clinical and socio‐demographic factors, melatonin, polypharmacy, post‐traumatic stress disorder, trauma

## Abstract

**Background:**

Clinical and demographic factors influencing prescribed sleep medication use in children and young people (CYP) have been studied inadequately. We aimed to examine the prevalence of prescribed sleep medication use among CYP experiencing mental health crises and its associations with clinical and demographic factors.

**Materials and Methods:**

Electronic medical records of CYP (*n* = 201) attending the Macarthur Safeguards Acute Mental Health Crisis Service (referred to as the Safeguards Service) in Sydney, Australia, were reviewed. Descriptive statistics and bivariate and multivariate logistic regression analyses were performed. Multivariate logistic regression was used to estimate the Adjusted Odds Ratio (AOR) with 95% confidence intervals, examining the associations between prescribed sleep medication use and clinical and demographic factors.

**Results:**

The prevalence of prescribed sleep medication use (e.g., melatonin, benzodiazepines) among CYP was 20.9%. Multivariate regression analysis indicated that CYP's length of stay with the Safeguards Service was positively associated with increased prescribed sleep medication use (AOR = 3.61, 95% CI: 1.19–10.94, *p* = 0.023). CYP who were diagnosed with trauma‐related psychiatric conditions tended to use more prescribed sleep medication use (AOR = 2.81, 95% CI: 1.09–7.19, *p* < 0.032). Moreover, multiple medication prescriptions (polypharmacy) were strongly associated with increased prescription of SM (AOR = 11.36, 95% CI: 4.46–28.95, *p* < 0.000).

**Conclusion:**

Prescribed sleep medication use is significant in CYP experiencing mental health crises. This requires careful consideration due to potential risks, drug interactions, and limited evidence of long‐term benefits. There needs to be a focus on addressing the underlying causes of sleep difficulties and exploring adjunct non‐pharmacological interventions, especially for those with trauma‐related psychiatric conditions, those who tend to have a longer length of stay and are prescribed multiple medications, including for sleep.

## Summary

**What is Known?:**

- Factors influencing prescribed sleep medication use have been well studied in adults, but evidence in children and young people (CYP) remains limited.
- There is a particular lack of research focusing on CYP presenting to acute mental health services, resulting in limited evidence to guide clinical management of sleep disturbances in this high‐risk population.

**What is New?:**

- By examining the associations between extended length of stay in mental health crisis services, trauma‐related diagnoses, and prescribed sleep medication use, our study contributes novel evidence to a limited and under‐researched area of the literature.
- The observed link between polypharmacy (prescribing multiple medications) and increased sleep medication use highlights the need for clinicians to carefully review medication regimens and consider evidence‐based, non‐pharmacological sleep interventions for CYP with sleep disturbance.

**What is Significant for Clinical Practice?:**

- Practitioners, including psychiatric mental health nurses, should proactively screen for sleep difficulties and review sleep medication use in CYP in mental health crisis, especially those presenting with trauma‐related diagnoses, polypharmacy, or extended stays, as these groups are at heightened risk of sleep medication reliance.
- Clinicians may consider prioritising early, trauma‐informed, non‐pharmacological sleep interventions (e.g. psychoeducation, behavioural and sleep hygiene strategies), while reserving sleep medications for short‐term use only when these approaches are ineffective or not feasible.

## 1 Introduction

Sleep disturbance is common in childhood (Krishna et al. 2023), and the prevalence is higher in children and young people (CYP) aged 5–17 years with psychiatric and neurodevelopmental disorders (El Halal and Nunes 2026). The prevalence is estimated to be 88% among CYP in mental health care in the United Kingdom (Halstead et al. 2021) and 65% in the Netherlands (van Tetering et al. 2025). The prevalence is also reported to be higher in Asian CYP with neurodevelopment disorders: 75%–80% in Thailand (Tippawanich et al. 2024) and 39.80%–50.4% in Japan (Kuki et al. 2024). In Australia, 42% of CYP have been reported as having a problem with their sleeping pattern (Rhodes et al. 2025). Sleep disturbances tend to persist over a long period and may lead to persistent insomnia (de Zambotti et al. 2018), which can cause clinically significant distress due to impairment in academic, behavioural, cognitive, educational or other areas of functioning (Arboledas et al. 2017). Persistent sleep disturbances are over‐represented in CYP experiencing emotional and behavioural problems, including depression, anxiety, mood, and emotional dysregulation (Liu et al. 2024), indicating the importance of non‐pharmacological strategies and the appropriate use of medication when the former is ineffective (Wei et al. 2020).

Peer‐reviewed studies of the prevalence of prescribed sleep medication use among CYP are growing, with a reported prevalence rate ranging from 17% to 50%. For example, a Swedish nationwide registered study found that among people with different age groups, including CYP (5–17 years), with attention deficit hyperactivity disorder (ADHD), 47.5% had been prescribed sleep medication, with 12% in those without ADHD, indicating the increased prevalence of sleep medication use in this clinical population, including CYP (Ahlberg et al. 2023). Few studies in the US and New Zealand reported prevalence estimates of sleep medication use in CYP. A US study among youth with diagnosed insomnia reported a prevalence of 37% (Bushnell et al. 2024), while a New Zealand study among CYP with autism or ADHD reported a prevalence of 25% (McLay et al. 2022). Further, an increase in prescription prevalence of benzodiazepines and other medications for sleep is being reported, with Hong Kong reporting an increase from 1.88% to 5.69% between 2000 and 2020 for 4–24 year olds (Kwok et al. 2025). In Australia, rates of sleep medication prescribing for CYP in primary care increased from 1.7% in 2011% to 5.9% in 2014% to 11.8% in 2018 (Klau et al. 2022), with more recent data indicating higher rates of use, such as 22.47% reported in 2023 (Glass et al. 2023). Taken together, the evidence suggests that sleep medication use is substantially higher among CYP with mental health and sleep disorders compared to general populations, with increasing prescribing trends observed over time in several settings.

However, the use of sleep medication raises legitimate concerns about its impact among CYP (Boafo et al. 2020). Many sleep medications, such as melatonin, benzodiazepines, and Z‐drugs, have limited information on their effects on CYP, including daytime drowsiness, dizziness, mood swings, irritability, hyperactivity, and decreased daytime functioning, which can negatively impact school performance and well‐being (Haendel et al. 2023). Moreover, sleep medications are often prescribed in the context of co‐occurring psychiatric conditions and in combination with other psychotropic medications, including antidepressants, antipsychotics, and stimulants (Bushnell et al. 2024). Such combinations may lead to polypharmacy that raises the risk of drug–drug interactions (Özbek et al. 2025), cumulative sedation, tolerance, dependence, and other adverse outcomes (e.g., cardiac arrhythmias and suicidal ideation) (Zito et al. 2021). Given these concerns, understanding the factors associated with sleep medication use among CYP is essential to guide appropriate prescribing, prioritise non‐pharmacological interventions when suitable, and adopt a targeted, risk‐informed approach.

There is extensive literature on the use of sleep medications in the adult population (Delpino et al. 2023), and in particular focusing primarily on the older age group and those with comorbid chronic diseases and sleep problems‐related comorbidities, highlighting polypharmacy, treatment appropriateness, psychosocial factors, and medication adherence (Zaidel et al. 2021). However, the number of studies on sleep medication use in CYP with mental health conditions is markedly lower compared to studies on adults. Available studies on CYP from Hong Kong (Kwok et al. 2025) and Norway (Oerbeck et al. 2020) indicate that older age is often associated with a higher prevalence of sleep medicine prescriptions. However, these studies did not include CYP attending acute mental health services. Increased sleep medication use was observed to be associated with female gender in Denmark, Norway and Sweden (Wesselhoeft et al. 2021). Unaffordability of behavioural interventions like cognitive behavioural therapy (CBT) has been reported as a key reason for medication use in Taiwan (Tsay et al. 2024), while marketing strategies have been reported as the reason for the rise of melatonin prescriptions among CYP in Norway (Stangeland et al. 2023). Thus, diverse factors have been attributed to the increase in sleep medication use among CYP with mental health issues internationally.

Existing Australian studies have mainly focused on predictors of psychotropic medication use (Wood et al. 2023) and management of insomnia in adults (Russell et al. 2023), while perspectives and experiences of Australian caregivers and community pharmacists have been reported in relation to paediatric melatonin use (Lee et al. 2024), behavioural strategies for managing sleep disturbances (Heussler et al. 2013) and clinical factors such as co‐occurring mental health conditions, stimulant use and parental mental health in the context of sleep difficulties (Sciberras et al. 2023) among CYP. Australian literature has also found that increased parental stress, child's age, and a diagnosis of ADHD had statistically significant relationships with increased use of melatonin (Glass et al. 2023). Another study involving CYP found that females were more likely to be long‐term benzodiazepine users compared to males (Woods et al. 2022). Collectively, these studies highlight growing but fragmented evidence on sleep and medication use in CYP.

However, these studies have not considered other demographic and clinical factors, which are plausibly linked to sleep medication use. In particular, housing insecurity and broader psychosocial context (e.g., family conflict, maltreatment, foster care) can interrupt sleep and increase dependence on sleep medications (Oerbeck et al. 2020). Evidence also demonstrates that poor sleep is related to self‐harm, and the use of sleep medications is common in CYP with self‐harm (King et al. 2019). CYP with trauma‐related diagnoses are more likely to report sleep problems (So et al. 2023), thus making them prone to use sleep medications (Barnett and Concepcion Zayas 2019). Polypharmacy is common in mental health crisis services, and a longer length of stay with mental health services indicates more complex mental health problems, suggesting greater exposure to sleep medications (Baeza et al. 2018). To our knowledge, there are no studies that have examined prescribed sleep medication use and its demographic and clinical factors among CYP receiving mental health crisis intervention and support, an inherently at high‐risk group for insomnia due to various causes and consequent polypharmacy, including sleep medication use (Ipsiroglu et al. 2022). This study aims to address this gap by examining the prevalence of sleep medication prescription and related demographic and clinical characteristics of CYP presenting to acute mental health crisis. The findings are expected to inform the management of this specific group, particularly about identifying the risks related to overprescription, drug interactions, and the long‐term implications of sleep medication use.

## 2 Materials and Methods

### 2.1 Study Design

This cross‐sectional study utilised an electronic medical records (EMR)‐based file audit of routinely collected data from consumers attending the Macarthur Safeguards Acute Mental Health Crisis Service (referred to as the Safeguards Service) and their parents and carers. We followed the Strengthening the Reporting of Observational Studies in Epidemiology (STROBE) reporting guideline for observational studies and applied the design‐specific STROBE Cross‐Sectional Checklist (Von Elm et al. 2007). This study is a part of the larger Safeguards project in New South Wales (NSW) (Eapen et al. 2023).

### 2.2 Study Setting

The study was conducted within the Safeguards Service in Sydney, Australia. The Safeguards Service is a novel community‐based outreach and acute mental health crisis intervention and supports for CYP and their families/caregivers for up to 6–8 weeks using a holistic approach (Eapen et al. 2023). The Safeguards service is delivered by a multidisciplinary team comprising child psychiatry, senior nursing, and allied health professionals with the necessary expertise to provide crisis assessment, specialist clinical care, and short‐term therapeutic interventions for CYP presenting with complex mental health needs and their families.

### 2.3 Sample Participants and Data Collection

Between March 2022 and December 2023, 282 CYP presenting in an acute mental health crisis service, Macarthur Safeguards Service, Sydney, Australia, were assessed for eligibility. Of these, 43 CYP who were actively engaged in a long‐term mental health treatment programme were excluded. A total of 239 CYP met the eligibility criteria; we included CYP aged 1–17 years, those who were experiencing an acute mental health crisis and receiving care from the service during the study period. Thus, 239 CYP were enroled, and they and/or their parents/carers provided data. Two research officers reviewed EMR data, extracted demographic and clinical variables and prescribed sleep medication information, and entered these into an Excel database; clinicians verified the extracted data. Then, 38 cases were excluded due to limited information on sleep medication use and related factors. Thus, a total of 201 participants with complete pre‐ and post‐intervention outcome measures were included in the final analytic sample.

### 2.4 Measures

#### 2.4.1 Explanatory Variables

Demographic variables included age (≤ 12 years, 13–18 years), gender (male, female, other), length of stay with the Safeguards Service (≤ 42 days, > 42 days), presenting with self‐harm (no, yes), having trauma‐related diagnoses (no, yes) and being prescribed multiple medications/polypharmacy (≥ 2 concurrent medications for ≥ 1 day) (no, yes). Trauma‐related diagnoses were identified as a key clinical factor due to their high prevalence in children and young people in mental health crisis care and their strong association with sleep disturbances (e.g., insomnia, nightmares) that may drive sleep medication use. In this study, trauma‐related diagnoses refer to psychiatric conditions arising from exposure to traumatic or highly threatening events, as defined by the International Classification of Diseases, eleventh revision (ICD‐11) (Maercker and Eberle 2022). The choice of the variables and categorisation was informed by prior literature (Barnett and Concepcion Zayas 2019; Glass et al. 2023; Ipsiroglu et al. 2022; King et al. 2019; Wood et al. 2023; Woods et al. 2022) and clinical relevance.

#### 2.4.2 Outcome Variable

“Prescribed sleep medication use” refers to the actual use of medications prescribed to manage sleep‐related difficulties (interrupted sleep, initial, middle or late insomnia). Commonly prescribed medications included melatonin and benzodiazepines, which were prescribed specifically for sleep difficulties in CYP during the study period. To ensure that medications were prescribed for sleep‐related indications, the indication was verified through a detailed review of EMR records, including medication orders, prescribing notes, and clinical progress notes, as well as clinicians' assessment of prescribing rationale. Only medications explicitly documented as being prescribed for sleep difficulties were classified as prescribed sleep medication use.

To further confirm actual use (e.g., that participants who were prescribed sleep medications actually took them), clinicians on the research team, who had no direct involvement in the care of these CYP, reviewed the EMR files, medication administration records, and doctors' notes. Actual use was confirmed when at least one of the following was documented: (a) medication administration records showing the medication was given; (b) patient or caregiver self‐report of taking the medication in progress notes; (c) follow‐up notes documenting clinical response, ongoing monitoring of the prescribed sleep medication; or (d) discharge summaries confirming medication use. All participants identified as prescribed sleep medications had at least one of these forms of documentation confirming actual use, further adding to the robustness of the data. The participants were then categorised as (a) no, and (b) yes. Other sleep medications, such as zopiclone and zolpidem, were not prescribed for the cohort during the study period and were therefore not included.

### 2.5 Data Analysis

To minimise potential bias arising from EMR records, we applied the above eligibility criteria, used standardised variable definitions, and conducted comprehensive data‐cleaning and verification procedures prior to analysis. Of the 239 eligible participants, 38 (15.9%) were excluded due to missing data on prescribed sleep medication use and two explanatory variables, such as presenting with self‐harm and being prescribed multiple medications. Missingness across individual variables was low among the remaining participants, and complete data were available for all covariates included in the adjusted regression models.

The statistical analysis was conducted at three levels. First, to summarize the socio‐demographic and clinical characteristics of the participants using descriptive statistics (e.g., frequencies, percentages, means, and standard deviations). Secondly, a bivariate analysis, specifically the chi‐square test, to examine the relationships between explanatory variables and sleep medication use among CYP. Thirdly, binary (crude) and multivariable (adjusted) logistic regression was performed to investigate the association between prescribed sleep medication use and key clinical and demographic factors. The adjusted models included age, gender, length of stay with the Safeguards Service, presenting with self‐harm, having trauma‐related diagnoses and being prescribed multiple medications (polypharmacy) as covariates. All explanatory variables were entered simultaneously as covariates in the adjusted logistic regression models. These covariates were selected a priori based on clinical relevance and prior literature indicating their association with sleep disturbance and medication use. Before conducting multivariable logistic regression, the variance inflation factor (VIF) was calculated to detect multicollinearity among explanatory variables, with a VIF value greater than 10 indicating significant multicollinearity. In the diagnostics for binary logistic regression, the absence of multicollinearity was confirmed by a Variance Inflation Factor (VIF) value of ≤ 1.60 for each included variable. The analyses were based on a complete case analysis, which means that only participants without missing data were included. All crude and adjusted logistic regression models included 201 participants. All statistical analyses were performed using the Statistical Package for Social Sciences (SPSS) software (Version 25.0). The statistical significance was set at the 5% level for all analyses.

### 2.6 Ethical Statement

On 7 March 2023, ethics approval for this study was obtained from the Human Research Ethics Committee (HREC) of the South Western Sydney Local Health District (Reference Number: 2022/ETH00808). The HREC granted a waiver of informed consent from parents/carers and/or assent from young people because this study involved no more than low risk and analysed routinely collected electronic medical record data obtained during standard clinical care. The current study was conducted in accordance with the Declaration of Helsinki (World Medical Association 2013).

## 3 Results

### 3.1 Participants' Socio‐Demographic and Clinical Characteristics

The sociodemographic characteristics of the participants in this study are presented in Table 1. A total of 201 CYP were included in the study. The majority of participants were adolescents (77.6%), while 22.4% were children. The study cohort consisted of 44.3% males, 51.2% females, and a small proportion (4.5%) identifying as non‐binary. Participants' average length of stay with the Safeguards Service was 48.72 days (standard deviation: 25.56). Around three‐fifths of the participants presented to the Safeguards Service with self‐harm (59.2%). Around one‐fourth of the participants had trauma‐related diagnoses (23.4%), while over two‐fifths were prescribed multiple (more than one) medications (43.3%).

**Table 1:** Sociodemographic and clinical characteristics of the participants (*N* = 201).

Characteristics	*n*	%
Age groups (years)		
Children (0–12 years)	45	22.4
Adolescents (13–18 years)	156	77.6
Gender		
Male	89	44.3
Female	103	51.2
Others/non‐binary	9	4.5
Length of stay with Safeguards Service (days)		
Mean = 48.72, SD = 25.56		
≤ 42 days	80	39.8
> 42 days	121	60.2
Presenting to Safeguards Service with self‐harm		
No	82	40.8
Yes	119	59.2
Having trauma‐related diagnoses		
No	154	76.6
Yes	47	23.4
Prescribed multiple medications		
No	154	56.7
Yes	87	43.3

### 3.2 Primary Psychiatric Diagnostic Profile of the Participants

Across the full sample (*N* = 201), internalizing disorders (including adjustment disorder, depressive disorder, anxiety disorder, selective mutism, eating disorder, and obsessive‐compulsive disorder) were most common (50.7%), followed by trauma‐related diagnoses (23.4%) (including acute stress reactions, post‐traumatic stress disorder and complex post traumatic stress disorders) and externalizing disorders (18.9%) (e.g., ADHD, conduct disorder, oppositional defiant disorder, and disruptive mood dysregulation disorder). Sleep medication use occurred in 20.9% of the cohort overall and varied by diagnostic category, with the highest prevalence among CYP with trauma‐related diagnoses (32.0%), compared with internalizing disorders (19.6%) and externalizing disorders (18.4%) (Table 2).

**Table 2:** Distribution of primary diagnostic categories and sleep medication use among CYP (*N* = 201).

Primary diagnostic categories	Total sample, *n* (%)	Sleep medication use, *n* (%)	No sleep medication use, *n* (%)
Development‐related diagnosis	5 (2.5)	0 (0.0)	5 (100.0)
Externalizing disorders	38 (18.9)	7 (18.4)	31 (81.6)
Internalizing disorders	102 (50.7)	20 (19.6)	82 (80.4)
Psychotic disorders	6 (3.0)	0 (0.0)	6 (100.0)
Trauma‐related diagnosis	47 (23.4)	15 (31.9)	32 (68.1)
Substance dependence	3 (1.5)	0 (0.0)	3 (100.0)
Total	201 (100.0)	42 (20.9)	159 (79.1)

### 3.3 Prevalence of Sleep Medication Use, Mental Health Conditions and Other Drugs Among Sleep Medication Users

Overall, more than one‐fifth of the participants were prescribed and used sleep medications (*n* = 42; 20.9%). Among these sleep medication users (*n *= 42), melatonin was overwhelmingly the most commonly prescribed agent (*n* = 41; 97.6%), with only one participant (2.4%) receiving a benzodiazepine. Table 3 details the prevalence of other medications used alongside sleep medications. It was found that antidepressants (e.g., Selective Serotonin Reuptake Inhibitors [SSRIs]) and Serotonin–Norepinephrine Reuptake Inhibitors [SNRIs]) were the most commonly used (54.2%), followed by antipsychotics (20.8%) and central nervous system stimulants (16.7%).

**Table 3:** Prevalence of other drugs among sleep medication users (*n* = 42).

Prevalence of other drugs	*n* (%)
Antidepressants (SSRIs, SNRIs)	26 (54.2)
Antipsychotics (second and first generation)	10 (20.8)
Central nervous system stimulants	8 (16.7)
Mood stabilisers	1 (2.1)
Supplements	1 (2.1)
Other drugs	2 (4.1)

### 3.4 Bivariate Associations Between Prescribed Sleep Medication Use and Its Factors

Table 4 shows the bivariate associations between various sociodemographic and clinical characteristics and the prevalence of sleep medication use in the study cohort (determined through chi‐square tests). CYP participants with a length of stay with the Safeguards Service of greater than 42 days were significantly more likely to use sleep medication as compared to participants who stayed for 42 days or less (26.4% vs 12.5%, *p* = 0.017). Sleep medication use was more prevalent among participants who were diagnosed with trauma than those who were not (31.9% vs 17.5%, *p* = 0.034). Similarly, CYP who were prescribed multiple medications were more likely to use sleep medication than individuals who were not (40.2% vs 6.1%, *p* < 0.001).

**Table 4:** Demographic and clinical characteristics of participants by overall and prescribed sleep medication use (*N* = 201).

Characteristics	Sleep medication use		
	No (%)	Yes (%)	a *p‐value*
Overall	79.1	20.9	
Age groups (year)			
Children (0–12 years)	73.3	26.7	0.280
Adolescents (13–18 years)	80.8	19.2	
Gender			
Male	78.7	21.3	0.761
Female	78.6	21.4	
Others/non‐binary	88.9	11.1	
Length of stay with Safeguards Service (days)			
Mean = 48.72, SD = 25.56			
≤ 42 days	87.5%	12.5%	**0.017**
> 42 days	73.6%	26.4%	
Presenting to Safeguards Service with self‐harm			
No	86.6%	13.4%	0.070
Yes	73.9%	26.1%	
Having trauma‐related diagnoses			
No	82.5%	17.5%	**0.034**
Yes	68.1%	31.9%	
Prescribed multiple medications			
No	93.9%	6.1%	**< 0.001**
Yes	59.8%	40.2%	

^a^ *p*‐values were derived from chi‐square tests.

### 3.5 Multivariable Logistic Regression Analysis of Factors of Prescribed Sleep Medication Use

Table 5 summarises the findings of the multivariable logistic regression model (adjusted and unadjusted odds ratios) used to identify factors associated with sleep medication use. Participants with a stay of 42 days or longer with the Safeguards Service had significantly higher odds of using sleep medication compared to those with shorter stays (AOR = 3.61; 95% CI: 1.19–10.94; *p* = 0.023) after adjusting for age, gender, presenting with self‐harm, having trauma‐related diagnoses and being prescribed multiple medications. Similarly, having trauma‐related diagnoses (AOR = 2.81; 95% CI: 1.09–7.19; *p* = 0.032) and being prescribed multiple medications (AOR = 11.36; 95% CI: 4.46–28.95; *p* < 0.001) were significantly associated with increased odds of sleep medication use after controlling for other factors. No significant associations were observed for age group, gender, or self‐harm presentation.

**Table 5:** Multivariable logistic regression model demonstrating the factors associated with prescribed sleep medication use among the participants (*N* = 201).

Independent variables	aCOR	95% CI	p‐value	bAOR	95% CI	*p*‐value
Age groups (year)						
Children (≤ 12 years)	Ref			Ref		
Adolescents (13–18 years)	0.66	0.31, 1.42	0.282	0.65	0.25, 1.67	0.37
Gender						
Male	Ref			Ref		
Female	1.00	0.51, 1.99	0.990	0.87	0.38, 2.00	0.74
Others	0.46	0.05, 3.91	0.478	0.24	0.02, 2.57	0.24
Length of stay with Safeguards Service (days)						
≤ 42 days	Ref			Ref		
> 42 days	2.52	1.16, 5.47	0.020	3.42	1.39, 8.42	**0.007**
Presenting to Safeguards Service with self‐harm						
No	Ref			Ref		
Yes	2.27	1.07, 4.48	0.071	2.22	0.90, 5.52	0.085
Having trauma‐related diagnoses						
No	Ref			Ref		
Yes	2.21	1.05, 4.63	0.036	2.77	1.09, 7.02	**0.032**
Prescribed multiple medications						
No	Ref			Ref		
Yes	10.29	4.28, 24.72	0.000	11.35	4.46, 28.85	**< 0.001**

Abbreviation: Ref = reference category.

^a^ Crude Odds Ratio.

^b^ Adjusted Odds Ratio.

## 4 Discussion

This present study is the first to explore the prevalence of prescribed sleep medication use and associated factors among CYP presenting with mental health crises in the Sydney region, Australia. Our findings revealed that the prevalence of sleep medication use among CYP was 20.9%. The findings of the logistic regression analysis revealed that CYP with a stay of 42 days or longer with the Safeguards Service team following a crisis presentation had significantly higher odds of using sleep medication compared to those with shorter stays. Having trauma‐related diagnoses and being prescribed multiple medications were found to be significantly associated with increased odds of sleep medication use.

We found that the prevalence of prescribed sleep medication use among CYP experiencing MH crises was 20.9%. This finding is broadly comparable to the prevalence reported by previous studies conducted among CYP in Norway (17%) (Oerbeck et al. 2020), New Zealand (25.0%) (McLay et al. 2021) and in Australia (22.5%) (Glass et al. 2023). However, only a few studies from Sweden (47.5%) (Ahlberg et al. 2023), Canada (40.0%) and the US (37.0%) (Bushnell et al. 2024) have reported such high rates of sleep medication use among CYP, whereas a study from Hong Kong found a much lower rate of 5.69% among young people (Kwok et al. 2025). These studies are different from the current study in many ways. For example, while our study was conducted among CYP with many mental health difficulties (e.g., internalising disorders, externalising disorders, and trauma‐related psychiatric conditions) in acute mental health crisis settings in Sydney, Australia, existing studies were conducted among CYP in residential care settings (Oerbeck et al. 2020), among CYP and young adults (< 25 years) (Bushnell et al. 2024; Kwok et al. 2025), those with autism (McLay et al. 2021), individuals with ADHD (Ahlberg et al. 2023), and parents (> 18 years) of children experiencing sleep disturbances (Glass et al. 2023). Thus, many factors, including different population groups, different mental health conditions, study settings, measurement tools, and types of sleep medication use, sampling variations, heterogeneity of study designs, and socio‐cultural differences, may explain the variations in sleep medication use among CYP globally.

The current study found that CYP with a more extended stay with the Safeguards Service had significantly higher odds of using sleep medication than those with shorter stays. This finding is clinically relevant, as participants with longer stays (> 42 days) were more likely to use sleep medication than those with shorter stays, suggesting that increased duration of care may reflect greater clinical complexity and unmet needs. Furthermore, family dysfunction, trauma‐related exposure, financial constraints and domestic violence often complicate the situation, requiring extended engagement with mental health services (Mesman et al. 2021). It is also important to consider that the observed associations with longer duration of stay, trauma‐related diagnoses, and polypharmacy may reflect underlying illness severity and clinical complexity rather than independent effects. CYP with more severe and complex mental health presentations are more likely to experience significant sleep disturbances, particularly in the context of trauma exposure and psychiatric comorbidity, where sleep problems are highly prevalent and related to greater symptom severity and poorer outcomes (Hyndych et al. 2025; Luo et al. 2022). Sleep disturbances are increasingly recognised as transdiagnostic features across psychiatric disorders and are bidirectionally associated with mental health severity, influencing symptom trajectories and clinical outcomes (Hyndych et al. 2025; Uccella et al. 2023). These disturbances contribute to increased clinical burden, including greater functional impairment and relapse risk, and often necessitate more intensive management approaches, including pharmacological interventions in more complex cases (Freeman et al. 2020). As such, these associations should be interpreted with caution and not assumed to be causal. We found no published studies in Australia and internationally, specifically examining the association between CYP's sleep medication use among those presenting in a mental health crisis.

Previous international studies have examined the type of sleep disturbances and related comorbidities, polypharmacy, and treatment suitability (Zaidel et al. 2021) as well as the use of behavioural and sleep medications among CYP (Larsson et al. 2023). Existing evidence also suggests that sleep disorders among CYP are associated with increased healthcare utilisation, including hospitalisations and service use, and may reflect greater clinical complexity requiring more intensive care (Adavadkar et al. 2024). Our study, by examining the association between extended stay in mental health crisis intervention as well as the link with trauma‐related diagnoses and increased prescription and use of sleep medication, has added to the limited body of literature in this area. Specifically, appropriate intervention for trauma‐related issues could potentially alleviate polypharmacy and decrease sleep medication use among CYP presenting in mental health crisis.

The reported associations between having trauma‐related diagnoses (PTSD and emerging borderline personality disorder) and increased sleep medication use are consistent with the finding that CYP with PTSD are more likely to be prescribed sleep medications (e.g., prazosin, guanfacine) (Keeshin et al. 2017), including for sleep disturbances, including nightmares and insomnia (Ferrafiat et al. 2020; Flores et al. 2024). The number of days of sleep medication use increased twofold in CYP whose parents had comorbid PTSD (Ahmed et al. 2020). This may suggest the need for family and interpersonal psychotherapeutic work (e.g., trauma‐focused cognitive behavioural therapy (TF‐CBT) for CYP and parents, both experiencing trauma and PTSD‐related issues, rather than resorting to longer duration of sleep medication use for symptom control. However, evidence indicates that TF‐CBT can be lengthy and CYP need to be out of crisis mode to be able to engage them in TF‐CBT meaningfully and effectively. This may indicate the necessity for sleep medications during mental health crises.

The observed associations of prescribing multiple medications with increased sleep medication use among CYP in our study are broadly similar to existing literature, suggesting that CYP with autism and ADHD who were prescribed melatonin had the highest prevalence of polypharmacy use in New Zealand (McLay et al. 2022). Our finding is also consistent with a Swedish study, indicating that dispensing melatonin was associated with concomitant dispensations of psychotropic medications (Kimland et al. 2021). The increased use of prescribed multiple medications, including sleep medications, highlights the need to also consider non‐pharmacological interventions, where appropriate, for CYP with sleep disturbances.

### 4.1 Strengths and Limitations

The present study has both strengths and limitations. To our knowledge, no other study has been conducted in Australia focusing on the use of sleep medication and its factors among CYP presenting in acute mental health crisis and hence adds to the limited available literature on the use of prescribed sleep medication and its factors among CYP. As this study is cross‐sectional, it provides a snapshot of prescribed sleep‐medication use among CYP and its associated factors; therefore, temporal ordering cannot be established, and causal inferences cannot be drawn. This limitation may bias estimates and should be considered when interpreting the observed associations. Furthermore, results are likely applicable to similar CYP attending mental health crisis services in comparable settings, but generalisability beyond the Safeguards Service should be interpreted with caution. Also, the sample size of this study was limited, and we did not collect qualitative data on sleep medication use and related factors among CYP. This highlights the need for further studies using a mixed‐methods design. Moreover, we included melatonin and benzodiazepines only, which were definitely prescribed for insomnia in CYP. The majority of prescribed sleep medication use in this cohort comprised melatonin, with minimal use of benzodiazepines. There potentially could be other CYP in our sample who were prescribed other medications, such as risperidone, quetiapine, or olanzapine, for sleep difficulties, which we did not include in our study. This was so as these medications were prescribed for a variety of other mental health conditions as well in this age group. Other sleep medications (e.g., zopiclone and zolpidem) were not identified in our cohort during the study period and were therefore not included. This indicates the need for further studies involving various sleep medications used for insomnia and other mental health conditions, including their long‐term side effects on CYP and those medications that may be prescribed for multiple purposes such as antipsychotics and alpha agonists. Notably, this study did not capture the use or timing of non‐pharmacological interventions for sleep (e.g., cognitive behavioural therapy or sleep hygiene strategies). As such, it is unclear whether prescribed sleep medications were initiated as first‐line treatment, used concurrently with non‐drug approaches, or prescribed following inadequate response to such interventions. Therefore, interpretations regarding clinical practice patterns should be considered with caution. Importantly, this represents a critical gap in the current evidence base. Future research should examine the sequencing, integration, and effectiveness of pharmacological and non‐pharmacological interventions for sleep among CYP presenting with acute mental health crises, including prospective and mixed‐methods designs to better inform clinical decision‐making.

### 4.2 Implications for Policy and Clinical Practice

This study highlighted a need for stakeholders and policy makers to collaborate to address the high prevalence of sleep medication use in CYP experiencing MH crises. It is particularly critical that policy makers develop strategies for CYP with trauma‐related diagnoses, prescribing multiple medications and extended programme length of stay, as they are at greatest risk for using sleep medications. Sleep difficulties are a complex issue in CYP with a bidirectional relationship with mental health issues that requires a coordinated, multi‐level strategy to be adequately addressed (Boafo et al. 2020). Policymakers, General Practitioners, psychologists, psychiatrists and non‐government organisations should be involved to create a multi‐disciplinary approach to sleep disorders that implements person‐centred strategies and intensive non‐pharmacological management focused on addressing the underlying causes of sleep difficulties. CYP and their families should be counselled on these strategies, including consistent sleep hygiene and routines, relaxation techniques, avoiding electronic devices and stimulating activities before bedtime, and a conducive sleep environment with minimal light and noise exposure (Ogundele and Yemula 2022). Clinicians, including psychiatric mental health nurses and other clinicians, should initiate such psychoeducation alongside behavioural interventions to reinforce and support these strategies to improve sleep. The use of sleep medications should ideally be short‐term and used when other measures are ineffective or not feasible.

There is a need to balance the risks of sleep medication use to treat sleep disorders with the possible benefits (Felt and Chervin 2014). CYP and their families should be extensively counselled on potential risks of sleep medication use, including potential cardiac side‐effects (Moon and Lee 2022). CYP in contact with mental health services would benefit from regular review of sleep as part of their psychological/psychiatric evaluation, with comprehensive non‐pharmacological management of sleep difficulties prior to consideration of pharmacological treatment strategies. We recommend close monitoring of those CYP who are taking sleep medications, intending to transition to non‐pharmacological sleep strategies as acute mental health crises resolve. This would help to ensure that timely and appropriate strategies are used to manage sleep disorders in this population, especially those with a trauma background, to minimise pharmacological interventions.

## 5 Conclusion

We found a high prevalence of sleep medication use among CYP presenting in acute mental health crisis in Australia. Our findings suggest that sleep medication use in CYP experiencing mental health crises requires careful consideration due to potential risks, drug interactions, and limited evidence of long‐term benefits. There needs to be a focus on addressing the underlying causes of sleep difficulties and prioritising non‐pharmacological interventions, especially for those with trauma‐related psychiatric conditions, having a longer length of stay, and using polypharmacy. Further research is needed to investigate the long‐term effects of sleep medication in CYP.

## Author Contributions

**Conceptualization:** Md Nazmul Huda; **Data curation:** Ingrid Bartrop; **Formal analysis:** Md Nazmul Huda; **Funding acquisition:** Valsamma Eapen, Rajeev Jairam; **Investigation:** Giles Barton, Rajeev Jairam, Md Nazmul Huda, Ingrid Bartrop; **Methodology:** Md Nazmul Huda; **Project administration:** Md Nazmul Huda; **Resources:** Md Nazmul Huda, Rajeev Jairam; **Software:** Md Nazmul Huda; **Supervision:** Valsamma Eapen, Rajeev Jairam, Md Nazmul Huda; **Validation:** Giles Barton, Rajeev Jairam, Ingrid Bartrop; **Writing – original draft:** Hasan Jamali, Mobarrat Monir, Avick Rahman, Anne‐Marie Schlesinger, Md Nazmul Huda; **Writing – review & editing:** Valsamma Eapen, Rajeev Jairam, Craig Andrews, Md Nazmul Huda.

## Funding

The authors have nothing to report.

## Ethics Statement

On 7 March 2023, ethics approval for this study was obtained from the Human Research Ethics Committee (HREC) of the South Western Sydney Local Health District (Reference Number: 2022/ETH00808). The HREC granted a waiver of informed consent from parents/carers and/or assent from young people because this study involved no more than low risk and analysed routinely collected electronic medical record data obtained during standard clinical care. The current study was conducted in accordance with the Declaration of Helsinki (World Medical Association 2013).

## Conflicts of Interest

The authors declare no conflicts of interest.

## AI Disclosure Statement

No generative artificial intelligence (AI) tools were used in the writing, editing, data analysis, or preparation of this manuscript. All content was developed solely by the authors.

## Data Availability

The data that support the findings of this study are available on request from the corresponding author. The data are not publicly available due to privacy or ethical restrictions.
